# Pre-hospital intranasal analgesia for children suffering pain: a rapid evidence review

**DOI:** 10.29045/14784726.2019.12.4.3.24

**Published:** 2019-12-01

**Authors:** Gregory Adam Whitley, Richard Pilbery

**Affiliations:** University of Lincoln: ORCID iD: https://orcid.org/0000-0003-2586-6815; Yorkshire Ambulance Service NHS Trust: ORCID iD: https://orcid.org/0000-0002-5797-9788

**Keywords:** administration, intranasal, child, emergency medical services

## Abstract

**Introduction:**

Pre-hospital analgesic treatment of injured children is suboptimal, with very few children in pain receiving analgesia. Studies have identified a number of barriers to pre-hospital pain management in children which include the route of analgesia administration. The aim of this review is to critically evaluate the pre-hospital literature, exploring the safety and efficacy of intranasal (IN) analgesics for children suffering pain.

**Methods:**

We performed a rapid evidence review, searching from inception to 17 December 2018, CINAHL, MEDLINE and Google Scholar. We included studies of children < 18 years suffering pain who were administered any IN analgesic in the pre-hospital setting. Our outcomes were effective pain management, defined as a pain score reduction of ≥ 2 out of 10, safety and rates of analgesia administration. Screening and risk of bias assessments were performed in duplicate. We performed a narrative synthesis.

**Results:**

From 310 articles screened, 23 received a full-text review resulting in 10 articles included. No interventional studies were found. Most papers reported on the use of intranasal fentanyl (INF) (n = 8) with one reporting IN ketamine and the other IN S-ketamine. Narrative synthesis showed that INF appeared safe and effective at reducing pain; however, its ability to increase analgesia administration rates was unclear. The effectiveness, safety and ability of IN ketamine and S-ketamine to increase analgesia administration rates were unclear. There was no evidence for IN diamorphine for children in this setting.

**Conclusion:**

Interventional studies are needed to determine with a higher confidence the effectiveness and safety of IN analgesics (fentanyl, ketamine, S-ketamine, diamorphine) for children in the pre-hospital setting.

## Background

According to McCaffery (1968, p. 95) ‘pain is whatever the experiencing person says it is, existing whenever he [sic] says it does’. The World Health Organization (2017) and Lohman, Schleifer and Amon (2010) mandate that countries must provide pain treatment medication as a core obligation under the right to health. Pain can have psychological, physical and social consequences which impact on quality of life (Lohman et al., 2010). Without effective pain treatment, children are at risk of developing post-traumatic stress disorder (Saxe et al., 2001; Sheridan et al., 2014).

The management of pain is complex, especially in children, as age, developmental level, cognitive and communication skills and associated beliefs must be considered (Srouji, Ratnapalan, & Schneeweiss, 2010). Pre-hospital analgesic treatment of injured children is ‘suboptimal’ (Samuel, Steiner, & Shavit, 2015), with very few children in pain receiving analgesia (Hennes, Kim, & Pirrallo, 2005; Lerner et al., 2014; Shaw, Fothergill, & Virdi, 2015; Swor, McEachin, Seguin, & Grall, 2005; Whitley & Bath-Hextall, 2017).

Studies have identified a number of barriers to pre-hospital pain management in children (Murphy et al., 2014; Williams, Rindal, Cushman, & Shah, 2012), which include route of analgesia administration, with the intranasal (IN) route proposed to overcome the challenge of cannulation in children. The aim of this review is to critically evaluate the pre-hospital literature exploring the safety and efficacy of IN analgesics for children suffering pain.

### Research question

This rapid evidence review (RER) aims to inform a paediatric pain research working group on the utilisation of the IN route to administer analgesia to children in the pre-hospital environment. The following research question was proposed: Are IN analgesics safe and effective at reducing pain in children within the pre-hospital environment?

### Objectives

The objectives of this RER are to:

- search and evaluate the literature relating to the management of paediatric pain in the pre-hospital environment using IN and other routes of administration; and
- present the findings to inform a paediatric pain research working group.

## Methods

### Rapid evidence review

This RER is based on the methodology outlined by Collins, Coughlin, Miller and Kirk (2015). RERs (also referred to as rapid evidence assessments or rapid reviews) are literature reviews that use methods to accelerate or streamline the traditional systematic review process (Ganann, Ciliska, & Thomas, 2010). As such, they are typically completed in compressed timeframes when compared to a systematic review.

### Inclusion and exclusion criteria

In order to identify relevant studies that address the research question, the PICOS (participants, intervention, comparator, outcomes, studies) acronym was used (Table 1).

**Table 1.** Summary of inclusion and exclusion criteria.

PICOS	Inclusion criteria	Exclusion criteria
Participants	Pre-hospital paediatric (< 18 years) patients who are in pain	Paediatric patients who are in hospital Patients 18 years or older
Intervention	Analgesia administered via the IN route	Analgesia administered via other routes unless reported as a comparator
Comparator	Analgesia administered via other routes	
Outcomes	Effectiveness (reduction in pain score) Safety (adverse/serious events) Administration rates	
Studies	RCTs, quasi-RCTs, prospective and retrospective observational studies and case series/reports	Editorials, position statements, letters, literature reviews, consensus statements and qualitative studies

IN = intranasal; RCT = randomised controlled trial.

#### Participants

The search was restricted to paediatric patients, but included adolescents, so an upper age cut-off of under 18 years of age was chosen (United Nations, 1989). There was no lower age limit. Since it is possible that there are differences in the management of paediatric pain between the pre-hospital and in-hospital setting, both in terms of the range of available analgesics and the personnel who are likely to be undertaking that administration, only pre-hospital studies were included.

#### Intervention

The IN route of administration (in theory) removes some of the barriers to administering analgesics to children (Murphy et al., 2014; Williams et al., 2012). Specifically, this route does not require the infant or child to be old enough to understand the administration, which is required for inhaled analgesics such as Entonox^®^ for example. In addition, it is not as invasive as the intravenous route, which can be difficult to achieve and can cause further distress and pain.

#### Comparator

Where studies included comparisons with drugs administered via routes other than the IN route, the review attempted to compare the reported results that relate to the outcomes specified in this review. Non-pharmacological interventions, such as distraction and presence of parents, were not included as a comparator due to the low level of documentation and subsequent lack of representation within the literature, often being reported as a limitation (Browne et al., 2016; Jennings, Lord, & Smith, 2015; Lord, Jennings, & Smith, 2017).

#### Outcomes

##### Effectiveness

Effective pain reduction was defined as a reduction in pain score of ≥ 2 out of 10 using the numeric pain rating scale, Wong and Baker FACES^®^ scale or Face, Legs, Activity, Crying and Consolability (FLACC) scale. This measure has been deemed the minimum clinically significant difference (Bailey, Daoust, Doyon-Trottier, Dauphin-Pierre, & Gravel, 2010; Bulloch & Tenenbein, 2002; Tsze, Hirschfield, von Baeyer, Bulloch, & Dayan, 2015).

##### Safety

Safety of IN analgesics (incidence of adverse events and serious incidents).

##### Administration rate

Where reported, overall rates of analgesic administration will be assessed.

#### Studies

Randomised controlled trials (RCTs), quasi-RCTs and prospective and retrospective observational studies were eligible for inclusion. There was no restriction on language, but results were limited to research on humans. Editorials, position statements, letters, literature reviews, case reports and consensus statements were not eligible, but the references cited in these publications were reviewed and relevant papers included. Literature reviews were excluded to maintain a higher threshold of study quality. Qualitative studies were excluded due time constraints, considering the complex nature of meta-synthesis and meta-integration.

### Search strategy

CINAHL and MEDLINE were accessed for the literature search, with grey literature searched via Google Scholar (the first 100 results were included from this search). An initial scoping search was conducted to identify appropriate keywords and MeSH headings. The final CINAHL/MEDLINE literature search query and Google Scholar search were run on 17 December 2018. Full details of the search query can be found in Supplementary 1.

### Study selection

A first pass of the search results was conducted independently by both authors, who screened the title and abstract against the inclusion/exclusion criteria to determine whether the papers might be suitable for inclusion. Once this was completed, the authors came together to address any disagreements about paper suitability. An independent arbiter was available for disagreements relating to inclusion that could not be resolved.

The full text of papers that made it through the first-pass process was obtained and independently reviewed (second pass) by both authors. Those that met the inclusion criteria were put forward for inclusion in the review. Full-text papers which were excluded at the second-pass stage were still screened for potentially relevant studies to inform the review.

### Critical appraisal

Papers that successfully made it past the second-pass process were critically appraised. The robustness of the evidence was determined by evaluating each paper against a list of criteria described by Collins et al. (2015). Separate lists of criteria were available for quantitative interventional and observational studies. Each criterion was given a score of 1–3 (1 being the lowest), and from these an overall critical appraisal score of 1–3 was awarded, based on the most commonly awarded score for each criterion. However, studies were not excluded from the review as a result of their risk of bias assessment given the anticipated low numbers of studies available. See Supplementary 2 for the risk of bias assessments.

### Data synthesis

Once the final papers for inclusion had been selected, a synthesis of the evidence was conducted. This consisted of:

1. describing the volume and characteristics of the evidence base;
2. utilising the synthesis to answer the research questions;
3. highlighting the implications of the findings; and
4. making recommendations for further research.

## Results

**Figure 1:**
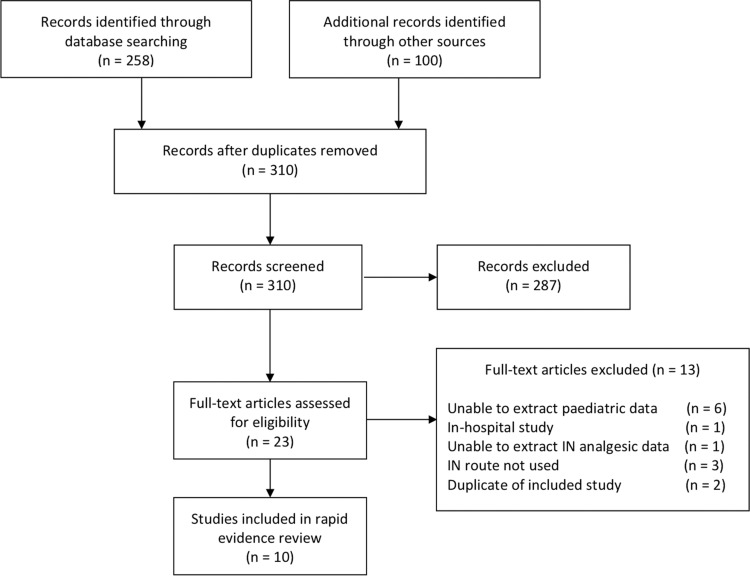
Figure 1. PRISMA flow diagram.

A total of 310 articles received a title and abstract screen; 23 of these received a full-text screen, which resulted in 10 articles included in this review (Figure 1). A summary of included studies can be found in Table 2.

**Table 2.** Summary of included studies.

**Study**	**Design**	**Country**	**Number of participants**	**IN analgesic**	**PICOS**	**Primary outcome measure**	**Key findings**
Pasquier, Eidenbenz, Dami, Ruffinen, & Hugli (2017)	Observational: retrospective	Switzerland	1156 (244 paediatrics)	Fentanyl	Participants: adults and children traumatically injured with isolated limb injury. Phenomena of interest: to examine pain management strategies and the time spent on-scene for analgesia provisions. Context: HEMS/mountain rescue.	Describe the different analgesic strategies used as well as the corresponding patient monitoring and medical co-treatments provided.	Effectiveness: n/a. Safety: no serious adverse events occurred. Authors were unable to exclude side effects that were either under-detected or under-reported. The findings support fentanyl as a safe analgesic, with minimal cardio-respiratory repercussions. Administration rate: the proportion of paediatric patients receiving fentanyl was significantly higher when given intranasally in comparison with the intravenous route (57% vs. 22%, p < 0.001). This difference also remained statistically significant after limiting the analyses to only the 71 cases where INF was used in first intention.
Murphy et al. (2017)	Observational: prospective	Ireland	94	Fentanyl	Participants: < 16 years, received INF during study period. Phenomena of interest: to describe the clinical efficacy and safety of INF when administered by advanced paramedics in the pre-hospital treatment of acute severe pain in children. Context: pre-hospital EMS system.	Does a single dose of INF at a dose of 1.5 microgram/kg (delivered in a 50 microgram/ml concentration), delivered through a MAD, produce an effective reduction in pain at 10 min after administration?	Effectiveness: a clinically effective reduction in the pain score was found in 78 children (83%; 95% CI 74–89%). Safety: no patient developed an adverse event as a result of INF. Administration rate: n/a.
Lord, Jennings, & Smith (2017)	Observational: retrospective	Australia	9833	Fentanyl	Participants: < 15 years, record of analgesic administration (methoxyflurane or morphine or fentanyl), pain recorded in secondary survey, or assessment, or patient complaint fields and initial pain severity score > 3. Phenomena of interest: the outcome of a change to practice guidelines that added INF and intramuscular morphine. Context: a large state-wide EMS.	The proportion of patients recorded as having a 2-point or greater reduction in pain severity score using an 11-point VNRS before and after a change of clinical practice guideline that added INF for the management of pain in children of any age.	Effectiveness: before the intervention, 88.1% (n = 3114) of children receiving analgesia had a reduction of pain severity of 2 or more points, with 94.2% (n = 5933) achieving this benchmark after intervention (p < 0.0001). The odds of a reduction in pain of 2 or more points increased by 1.01 per month immediately before the intervention and 2.33 after intervention (< 0.0001). Safety: n/a. Administration rate: n/a.
Browne et al. (2016)	Observational: retrospective	United States	7340	Fentanyl	Participants: < 18 years, injured children. Phenomena of interest: determine the change in frequency of documented pain severity assessment and opiate administration among injured paediatric patients in three EMS agencies after adoption of best practice recommendations. Context: pre-hospital urban EMS system.	Frequency of pain severity assessments and the documented administration of opioid analgesia before and after adoption of best practice recommendations.	Effectiveness: n/a. Safety: n/a. Administration rate: there was a difference in INF administration rates before (27% [n = 45]) and after (17% [n = 32]) protocol changes (p = 0.02). Opiate administration to eligible patients across study sites regardless of documentation of pain severity was 156/3089 (5%) before protocol changes and 175/3509 (5%) after (p = 0.97).
Lord, Jennings, & Smith (2016)	Observational: retrospective	Australia	38,167	Fentanyl	Participants: < 15 years; and pain in secondary survey, or pain in assessment, or pain in patient complaint, or pain score > 0. Phenomena of interest: to describe paramedic assessment and management of pain in children. Context: a large state-wide EMS.	To describe the prevalence and nature of pain in children using a major Australian EMS.	Effectiveness: for those receiving fentanyl (95.6% IN route): Initial pain score median (IQR): 8 (6–9). Pain score change median (IQR): 5 (3–7). Safety: n/a. Administration rate: 3274 (8.6%) children received fentanyl with 95.6% administered via the IN route.
Karlsen et al. (2014)	Observational: prospective	Denmark	903 (63 children < 18 years)	Fentanyl	Participants: adults and children over eight years (or > 30 kg), received INF for severe pain caused by orthopaedic or abdominal conditions, or ACS refractory to GTN. Phenomena of interest: the safety profile and apparent analgesic effect of INF. Context: pre-hospital EMS system.	Occurrence of adverse effects.	Effectiveness: in patients < 18 years of age, median reduction in pain score was 4 (IQR, 2–5), with 87% receiving clinically relevant reductions (pain score reduction of ≥ 2 points). Safety: 39 potential adverse events in 36 patients, none of which were serious (figures for children < 18 years not stated). Administration rate: n/a.
O’Donnell et al. (2013)	Observational: retrospective	United States	233	Fentanyl	Participants: < 16 years, trauma patients. Phenomena of interest: to examine the effect of introducing the MAD on analgesia administration as an alternative to intravenous fentanyl delivery in paediatric trauma patients. Context: pre-hospital EMS system.	Appropriateness of fentanyl administration as determined by two ED physicians.	Effectiveness: n/a. Safety: n/a. Administration rate: no statistically significant difference in the rate of fentanyl administration between the pre-MAD (30.4%) and post-MAD (37.8%) groups.
Johansson et al. (2013)	Case series	Sweden	9 (6 children < 18 years)	S-ketamine	Participants: adults and children, traumatic injuries where vascular access was foreseen or proven to be problematic (1 or 2 missed attempts). Phenomena of interest: describe experiences of using nasal S-ketamine for pre-hospital analgesia in nine especially challenging cases during two winter seasons (2010–2012). Context: HEMS/mountain rescue.	Median reduction in pain score before and 5 to 10 minutes after administration of nasal S-ketamine.	Effectiveness: VAS-score decreased from a median of 10 (IQR 7.5–10) to 3 (IQR 2–4), p = 0.0018. For children < 18 years (n = 6) decrease from a median of 10 (IQR 8–10) to 2 (IQR 1–3). Safety: side effects in these nine cases were few and non-serious. Administration rate: n/a.
Reid, Hatton, & Middleton (2011)	Case report	Australia	1	Ketamine	Participants: paediatric burn patient. Phenomena of interest: report the pre-hospital use of IN ketamine in a paediatric burns case. Context: pre-hospital EMS system.	Symptom relief.	Effectiveness: although formal pain scoring was not performed in the case described, a satisfactory level of analgesia and anxiolysis appeared to be achieved sufficient to allow the comfortable application of a burns dressing and patient transport. Safety: n/a. Administration rate: n/a.
Bendall, Simpson, & Middleton (2011)	Observational: retrospective	Australia	3312	Fentanyl	Participants: paediatric patients (5–15 years) with moderate to severe pain (VNRS-11 ≥ 5) who were treated by EMS with intravenous morphine, INF or inhaled methoxyflurane either alone or in combination. Initial and final pain scores recorded. Phenomena of interest: to compare the effectiveness of intravenous morphine, INF and inhaled methoxyflurane for managing moderate to severe pain in paediatric patients. Context: pre-hospital EMS system.	‘Effective analgesia’ defined as a reduction in pain score of ≥ 30% using the VNRS-11.	Effectiveness: all analgesic agents were effective in the majority of patients (87.5%, 89.5% and 78.3% for morphine, fentanyl and methoxyflurane, respectively). For those receiving INF alone, initial median (IQR) pain was 8 (7–10) with a median (IQR) pain score difference of 5 (3–7). There was evidence that methoxyflurane was less effective than both morphine (OR 0.52; 95% CI 0.36–0.74) and fentanyl (OR 0.43; 95% CI 0.29–0.62; p < 0.0001). There was no clinical or statistical evidence of difference in the effectiveness of fentanyl and morphine in this population (OR 1.22; 95% CI 0.74–2.01). There was no evidence that combination analgesia was better than either fentanyl or morphine alone. Safety: n/a. Administration rate: n/a.

ACS = acute coronary syndrome; CI = confidence interval; ED = emergency department; EMS = emergency medical service; GTN = glyceryl trinitrate; HEMS = helicopter emergency medical service; IN = intranasal; INF = intranasal fentanyl; IQR = interquartile range; MAD = mucosal atomisation device; OR = odds ratio; VAS = visual analogue scale; VNRS = verbal numeric rating scale.

Ten papers were included in this review and none were high-quality interventional trials. There were eight observational studies, one case series and one case report that met the inclusion criteria. These papers reported on the use of three IN analgesics: fentanyl (n = 8), S-ketamine (n = 1) and ketamine (n = 1). Seven studies reported on the effectiveness, four on the safety and four on the administration rate of IN analgesics administered to children (< 18 years) in the pre-hospital setting. Papers were deemed to have a ‘low risk of bias’ (n = 3), ‘moderate risk of bias’ (n = 5) and ‘high risk of bias’ (n = 2). The relevance of these studies to the research question, target population and outcome measure was deemed ‘high’ (n = 6) and ‘low’ (n = 4).

### Risk of bias and relevance to study question

Bendall, Simpson and Middleton (2011) and Lord, Jennings and Smith (2016, 2017) were deemed to have a ‘low risk of bias’ (see Supplementary 2) and were highly relevant to the research question, target population and outcome measure. Murphy et al. (2017), Karlsen et al. (2014), Pasquier, Eidenbenz, Dami, Ruffinen and Hugli (2017) and O’Donnell et al. (2013) were deemed at ‘moderate risk of bias’, with Murphy et al. (2017) and O’Donnell et al. (2013) being highly relevant and Karlsen et al. (2014) and Pasquier et al. (2017) less relevant to the target population. Johansson et al. (2013) and Reid, Hatton and Middleton (2011) were deemed at ‘high risk of bias’, with poor relevance to the target population and outcome measure.

### Effectiveness

#### Intranasal fentanyl

Murphy et al. (2017) reported on the use of intranasal fentanyl (INF) in children < 16 years (n = 94) and found that a clinically effective reduction in pain score occurred in 78 children (83%; 95% confidence interval (CI) 74–89%).

Lord et al. (2017) reported on the implementation of INF in children < 15 years (n = 9833) and found that before the intervention, 88.1% (n = 3114) of children receiving analgesia had a reduction of pain severity of 2 or more points, with 94.2% (n = 5933) achieving this benchmark after intervention (p < 0.0001). The odds of a reduction in pain of 2 or more points increased by 1.01 per month immediately before the intervention and 2.33 after intervention (p < 0.0001).

Lord et al. (2016) reported on the use of INF in children < 15 years (n = 38,167) and found that for those receiving fentanyl (95.6% of the study population received this via the IN route), the initial median pain score was 8 (interquartile range (IQR) 6–9) with a median pain score change of 5 (IQR 3–7).

Karlsen et al. (2014) reported on the use of INF in adults (n = 840) and children over eight years (n = 63) and found that in those aged < 18 years, the median reduction in pain score was 4 (IQR 2–5), with 87% achieving clinically relevant reductions (defined as a pain score reduction of ≥ 2 points out of 10).

Bendall et al. (2011) reported on the use of INF in children aged 5–15 years (n = 3312) and found that 89.5% of patients who received INF achieved effective pain reduction (defined as a reduction in pain score of ≥ 30% using the 11-point verbal numeric rating scale, VNRS-11). For those receiving INF alone, the initial median pain score was 8 (IQR 7–10) with a median pain score difference of 5 (IQR 3–7). There was evidence that methoxyflurane was less effective than fentanyl (odds ratio (OR) 0.43; 95% CI 0.29–0.62; p < 0.0001), but no clinical or statistical evidence of difference in the effectiveness of fentanyl and morphine in this population (OR 1.22; 95% CI 0.74–2.01). There was no evidence that a combination of analgesics was better than either fentanyl or morphine alone.

#### Intranasal ketamine/S-ketamine

Ketamine, or racemic ketamine, is a mixture of optical isomers. S-ketamine is a purer, single isomer of ketamine with twice the anaesthetic and analgesic potency of racemic ketamine (Doenicke, Kugler, Mayer, Angster, & Hoffmann, 1992).

Johansson et al. (2013) reported on the use of IN S-ketamine in adults (n = 3) and children (n = 6) in an alpine setting, and reported that for those aged < 18 years (n = 6) median pain scores reduced from 10 (IQR 8–10) to 2 (IQR 1–3).

Reid et al. (2011) reported on the use of IN ketamine in one paediatric patient suffering a thermal injury and found that a satisfactory level of analgesia and anxiolysis (reduction of anxiety) was achieved, allowing the comfortable application of a burns dressing and patient transport.

Bendall et al. (2011), Karlsen et al. (2014), Lord et al. (2016, 2017) and Murphy et al. (2017) all reported high levels of effective pain management when using INF, suggesting that INF is effective at reducing pain in children within the pre-hospital setting.

Johansson et al. (2013) and Reid et al. (2011) also reported effective pain management via the IN route, although with S-ketamine and ketamine, respectively. However, the high risk of bias and low relevance with respect to this review indicates that further research is required to assess the effectiveness of IN S-ketamine/ketamine for the treatment of pain in children in the pre-hospital setting.

### Safety

#### Intranasal fentanyl

Pasquier et al. (2017) reported on the use of INF in adults (n = 912) and children (n = 244) and found no serious adverse events, although, as a retrospective review, it has to be acknowledged that side effects could have been under-detected and/or under-reported. The authors concluded that fentanyl is a safe analgesic, with minimal cardio-respiratory repercussions. Karlsen et al. (2014) reported 39 potential adverse events in 36 patients, none of which were serious. However, it was not possible to determine the proportion of adverse events that occurred in children in this study. Finally, Murphy et al. (2017) recorded no adverse events during their study (n = 94).

#### Intranasal ketamine/S-ketamine

Johansson et al. (2013) did not explicitly quantify all side effects, although they did confirm that no patients had airway compromise. Three did experience vertigo (not specified whether adult or child) and ‘many’ complained of the taste.

### Administration rate

#### Intranasal fentanyl

Pasquier et al. (2017) found that the proportion of paediatric patients receiving analgesia was significantly higher when INF was available in comparison with the intravenous route only (57% vs. 22%, p < 0.001). This difference also remained statistically significant after limiting the analyses to only the 71 cases where INF was used as the first analgesic route and drug.

Browne et al. (2016) reported on the use of INF in children < 18 years (n = 7340) before and after treatment protocol amendments aimed at improving paediatric pain management. They found a reduction in INF administration rates from 27% (n = 45) before the protocol change, to 17% (n = 32) after (p = 0.02). In addition, there was no increase in opiate administration to eligible patients across study sites, regardless of documented pain severity. This remained at 5% throughout the study period.

The IN route was the most common as reported by Lord et al. (2016), with 95.6% of children < 15 years of age who were administered fentanyl receiving it via the IN route. In contrast, O’Donnell et al. (2013) reported on the use of fentanyl in children < 16 years (n = 233) before and after the introduction of a mucosal atomisation device (MAD) and found no statistically significant difference in the rate of fentanyl administration between the pre-MAD (30.4%) and post-MAD (37.8%) groups.

## Discussion

### Effectiveness

INF appears to be effective at reducing pain in children in the pre-hospital setting. The effectiveness of IN S-ketamine/ketamine could not be established given the low numbers of patients and poor quality of the included studies. There was no evidence of the effectiveness of other IN analgesics such as diamorphine for children in the pre-hospital setting.

### Safety

Overall, none of the included papers reported any serious adverse effects from analgesics administered via the IN route. INF appeared safe, but the poor relevance of Karlsen et al. (2014) and Pasquier et al. (2017) and the small sample size of Murphy et al. (2017) limit the strength of this finding. The safety of IN ketamine could not be established with any confidence, given the high risk of bias and poor relevance, compounded by a small sample size overall (n = 7) (Johansson et al., 2013; Reid et al., 2011).

Evidence for the safety of INF was limited, but suggested it was safe, with some minor but no serious side effects reported. The safety of IN S-ketamine could not be established with any confidence. There was no evidence to determine the safety of IN ketamine or diamorphine for children in the pre-hospital setting.

### Administration rates

Evidence regarding the administration rates of INF were conflicting and therefore could not be established. However, the studies from the United States appear to be outliers in terms of lower than expected paramedic utilisation of IN routes for paediatric analgesia. There was no evidence to determine the administration rates of IN S-ketamine/ketamine or diamorphine for children in the pre-hospital setting.

### Strengths and limitations

The lack of interventional studies limits the quality of data available for synthesis, meaning we were not able to determine causation. We were unable to determine whether INF causes effective pain management, so can only conclude that it appears to be effective.

The rapid nature of this review meant that we only searched a limited number of sources and did not search trial registries or perform forward/backward citation tracking; therefore, there is a possibility that some articles have been missed. We did not determine the confidence in the cumulative evidence.

We believe a strength of this review is the limitation to the pre-hospital context. This allows for simpler analysis and interpretation and the development of clear recommendations for future research in this setting.

### Recommendations for future research

Interventional studies are needed to determine with a higher confidence the effectiveness and safety of IN analgesics (fentanyl, ketamine, S-ketamine, diamorphine) for children in the pre-hospital setting.

## Conclusion

INF appeared effective and safe, but its ability to increase analgesia administration rates was unclear. The effectiveness, safety and ability of IN ketamine/S-ketamine to increase analgesia administration rates could not be determined. There was no evidence relating to the use of IN diamorphine for children in the pre-hospital setting. Further research is required, preferably utilising an interventional approach, to improve the quality of evidence.

## Author contributions

Both authors made substantial contributions to the conception and design of this review; the acquisition, analysis and interpretation of data; drafting the work and revising it critically for important intellectual content; and final approval of the version to be published. They agree to be accountable for all aspects of the work in ensuring that questions related to the accuracy or integrity of any part of the work are appropriately investigated and resolved.

## Conflict of interest

GAW declares no conflict of interest. RP is the editor-in-chief of the *British Paramedic Journal* but had no editorial control over this publication.

## Ethics

Not required.

## Funding

None.
